# Toward Control? The Prospects and Challenges of Typhoid Conjugate Vaccine Introduction

**DOI:** 10.1093/cid/ciz483

**Published:** 2019-10-15

**Authors:** Megan E Carey, Zoey I Diaz, Martin Broadstock, Roderick Bailey, Adwoa D Bentsi-Enchill, Heidi J Larson

**Affiliations:** 1 Enteric and Diarrheal Diseases, Global Health, Bill & Melinda Gates Foundation, Seattle, Washington; 2 Immunology and Vaccines, Medical Research Council, London; 3 Wellcome Centre for Ethics and Humanities, University of Oxford, United Kingdom; 4 Department of Immunization, Vaccines and Biologicals, World Health Organization, Geneva, Switzerland; 5 Department of Infectious Disease Epidemiology, London School of Hygiene and Tropical Medicine, United Kingdom; 6 Department of Health Metrics and Evaluation, University of Washington, Seattle

**Keywords:** typhoid control, typhoid conjugate vaccine, water and sanitation, public awareness, vaccine acceptance

## Abstract

With a newly World Health Organization (WHO)–prequalified typhoid conjugate vaccine (TCV), Gavi funding for eligible countries, and a WHO policy recommendation for TCV use, now is the time for countries to introduce TCVs as part of an integrated typhoid control program, particularly in light of the increasing burden of antimicrobial resistance. Continued vaccine development efforts will lead to secure supply of low-cost vaccines, and ongoing vaccine studies will provide critical vaccine performance data and inform optimal deployment strategies, in both routine use and in outbreak settings. TCV programs should include thoughtful communication planning and community engagement to counter vaccine hesitancy.

This is a historic moment for typhoid control. Despite continuing gaps, we now have a more refined understanding of the burden of typhoid fever, as well as improved tools available to reduce the burden dramatically. Recent multicenter surveillance studies in Africa and Asia have also improved understanding of the age and geographic distribution of typhoid, demonstrating that it is an illness that affects young children as well as school age children living in both urban and rural settings [1–3]. These and other epidemiological data informed the recommendations by the World Health Organization (WHO) Strategic Advisory Group of Experts on Immunization in October 2017 on the use of typhoid conjugate vaccine (TCV) for typhoid control [4]. Shortly after this recommendation was issued, Bharat Biotech’s (Hyderabad, India) Typbar-TCV (composed of Vi polysaccharide conjugated to tetanus toxoid) became the first TCV to obtain WHO prequalification [5]. Gavi, the Vaccine Alliance, a public-private partnership that channels financing and works with other alliance partners to support lower-income countries for equitable use of vaccines, approved funding support for TCV use in Gavi-eligible countries in November 2017 [6]. The Typhoid Vaccine Acceleration Consortium (TyVAC), a partnership between the University of Maryland, Oxford University, and PATH, is assessing the efficacy of this vaccine at field sites in Bangladesh, Nepal, and Malawi [7].

Since the first WHO recommendation in 2000 for the unconjugated Vi polysaccharide (Vi-PS) and live-attenuated Ty21a vaccines, considerable progress in vaccination as a typhoid control strategy has been realized. The updated 2018 WHO policy on typhoid vaccination includes a first-time recommendation for the routine use of TCVs, including in children <2 years of age in countries with a high burden of disease and/or high rate of antimicrobial-resistant *Salmonella* Typhi; these children would not have been protected by either the Vi-PS or Ty21a vaccine. A single intramuscular dose of TCV is recommended for primary vaccination of infants and children from 6 months of age and adults up to 45 years of age, with an option for catch-up campaigns in children up to 15 years of age [8]. Furthermore, the use of TCV is recommended in preference to the Vi polysaccharide and Ty21a vaccines in both routine immunization programs and in response to confirmed outbreaks.

Historical precedents in many industrialized countries teach us that typhoid control is feasible through establishment and continued maintenance of water and sewage systems that ensure that food and drinking water are separated from human fecal contamination [9, 10]. However, the challenge of making sustainable improvements to water and hygiene infrastructure is yet to be overcome in many endemic countries and requires significant time and investment, particularly given additional demands on the existing water supply caused by rapid urbanization and climate change [10]. Yet another challenge, and one that makes a strong case for prioritizing parallel short-term control measures, is the increasing prevalence of antimicrobial-resistant strains of *S.* Typhi [11–13].

## FUTURE AREAS OF FOCUS: NEW VACCINES AND SURVEILLANCE

Reducing the global burden of typhoid is a focus of both international health organizations and major global health funders. A central tenet of this focus is optimal deployment of vaccines in endemic regions, and critical to its success is sustainable, secure vaccine supply [6, 8, 14, 15]. There is a robust pipeline of typhoid conjugate vaccine candidates in clinical development [16]. Among the more advanced candidates, is a TCV composed of Vi polysaccharide conjugated to diphtheria toxoid, developed by the International Vaccine Institute, which is undergoing clinical development through a technology transfers to SK Bioscience in Korea, PT BioFarma in Indonesia, and Incepta Vaccine Ltd. in Bangladesh [16]. Another candidate, TCV with a non-toxic mutant of diptheria as carrier protein (Vi-CRM197) is being developed by Biological E Ltd. in India through a technology transfer from the Novartis Vaccines Institute for Global Health (now GSK Vaccines Institute for Global Health) [16]. The TCV candidates from SK Bioscience, PT BioFarma and Biological E Ltd. are currently being evaluated in clinical studies in the Phillipines, Indonesia, and India respectively [16].

While the recent prequalification of the Bharat Biotech TCV and the pipeline of TCV candidates augur well for a healthy vaccine market, there continues to be a need to understand the impact and operational feasibility of large-scale TCV deployment strategies. The TCV studies being conducted under the leadership of TyVAC will provide field efficacy data, outcomes that are made more critical by the fact that national licensure of the vaccine was based on immunogenicity data [7]. As with all new vaccine introductions, as countries begin to introduce TCVs into routine use, it will be important to assess their impact on disease and to track coverage and sustainability of routine TCV use over time. There are also important operational research questions that need to be addressed about how best to target TCV catch-up campaigns to maximize health impact. Additionally, as typhoid is a disease with outbreak potential, understanding how, when, and where to deploy TCVs to prevent and/or halt the spread of an outbreak continues to be an important issue.

To improve monitoring of the disease burden, the WHO recently released surveillance standards for *S*. Typhi and other invasive salmonelloses [17] and recommended laboratory-based surveillance as a minimum standard. Because blood-culture–based surveillance is time and resource intensive and has low sensitivity [18], there is a need to understand the role of additional methods to assess enteric fever burden in resource-limited settings. Initially, new methods should be tested and validated at sites where blood culture surveillance has already been established. New methods could include environmental surveillance data, gene expression signatures [19], and serological markers of current or past infection [20]. Once validated, the outputs of such assessments could support country decision making on TCV introduction, as well as determine optimal vaccination strategies. Ideally, these efforts would be incorporated into integrated surveillance systems encompassing additional pathogens.

The ongoing rise of antimicrobial resistance (AMR) in *S.* Typhi is a major factor contributing to the need for vaccine introduction and increased surveillance [21]. The US Centers for Disease Control and Prevention lists S.Typhi, with ongoing emergence of multi-drug resistant strains as a ‘serious threat’ [22]. In 2017, the WHO included *Salmonella* species in its list of antibiotic-resistant “priority pathogens” to guide and promote research and development of new antibiotics to address the global growth of AMR [23]. The emergence of extensively drug-resistant *S.* Typhi isolates with resistance against chloramphenicol, ampicillin, trimethoprim-sulfamethoxazole, fluoroquinolones, and third-generation cephalosporins enhances the threat of infections with limited treatment options [24]. The widespread uptake of TCVs, with their improved immunogenicity in children and longer duration of protection, has potential to combat AMR in typhoid endemic areas, provided sufficient vaccine coverage levels are achieved, particularly in groups at greatest risk of disease. As such, TCVs could both reduce the number of circulating drug-resistant strains of *S*. Typhi and reduce the overall incidence of febrile illnesses, thereby reducing the need for and exposure to antibiotics.

## DEPLOYING NEW VACCINES: PUBLIC ACCEPTANCE AND IMPACT ON GLOBAL SECURITY

Increasing global interconnectedness and the resultant growing risk of infectious disease spread across wide geographies make it imperative that new vaccines and improved surveillance are accompanied by a better understanding of local perceptions about specific vaccines and the diseases that they aim to prevent.

In 2019, the WHO named “vaccine hesitancy,” as one of 10 global health threats, alongside Ebola, AMR, and climate change [25]. Global surveys show that, on average, populations’ belief in the importance of vaccines and acceptance of routine vaccination is relatively high, although highly uneven across different settings [26, 27]. There is concern that people’s confidence in vaccines is eroding and can result in falling vaccination rates. The large number of measles outbreaks in recent years illustrates this in part (access to vaccines being another important cause in many regions), with Europe alone having nearly 85 000 cases of measles in 2018 [28]. The introduction of new vaccines impact vaccine coverage in different ways; in some cases improving coverage, and in others disrupting immunization services. For example, in the Philippines, it has been demonstrated that reduced public confidence due to safety scares around one new vaccine can spill over to create reluctance around and refusal of other vaccines. This was particularly evident by the increase in measles vaccination refusals, which resulted in large-scale measles outbreaks [29]. These are important lessons to bear in mind ahead of the introduction of the newly available TCV. The importance of community engagement, from the early stages of considering the introduction of a new vaccine or new immunization initiative, is well established, as are the consequences of not adequately engaging stakeholders. Vaccine program challenges in the context of conflict and insecurity, which the global polio eradication initiative has faced in the last mile of a multidecade effort, is another area ripe with lessons learned [30].

Similar to many vaccine-preventable diseases, typhoid thrives in conditions of sociopolitical breakdown. Past conflicts have exposed refugees in multiple locations to the catastrophic impact of infected food and water sources. For example, crowding and degraded living conditions, including poor sanitation and hygiene, have contributed to typhoid outbreaks in Bosnia-Herzegovina Ivory Coast, Rwanda, Tanzania, South Sudan, North Darfur, and Syria [31–34]. In most instances, these outbreaks occurred in refugee camps characterized by dense population, vulnerable infrastructures, and strained resources. In addition to detection and treatment programs, vaccination has the potential to contribute significantly to successful disease control in such circumstances [35]. More difficult to address can be social problems caused or aggravated by outbreaks. In conflict areas, these can include rumors, stigmatization, and conflicts within and between vulnerable populations. In situations like these, successful disease control requires an effective and rapid interplay between disease surveillance, medical intervention, and culturally appropriate information policies.

## CONCLUSIONS

With the availability of improved typhoid disease burden data and low-cost TCVs, countries have a unique opportunity to implement TCVs as part of an integrated typhoid control program. Given the increasing threat of AMR, and the longer time horizon of improving water and sanitation infrastructure, TCVs are a critical tool in both immediate and long-term typhoid control efforts. Routine use of TCVs in endemic countries has considerable potential to reduce the burden of disease, and TCVs have a vital role to play in outbreak control, particularly in vulnerable populations. It is important to implement lessons learned from other vaccine introductions and to represent the full societal value of TCV programs through thoughtful advocacy and communications planning to counter vaccine hesitancy.

## Notes

***Supplement sponsorship.*** This supplement has been funded by the New Venture Fund.

***Potential conflicts of interest.*** H. J. L. has received grants from GlaxoSmithKline, outside the submitted work. All other authors report no potential conflicts of interest. All authors have submitted the ICMJE Form for Disclosure of Potential Conflicts of Interest. Conflicts that the editors consider relevant to the content of the manuscript have been disclosed.
